# Hodgkin Lymphoma and Castleman Disease: When One Blood Disease Can Hide Another

**DOI:** 10.1155/2017/9423205

**Published:** 2017-01-18

**Authors:** L. Filliatre-Clement, H. Busby-Venner, C. Moulin, G. Roth-Guepin, A. Perrot

**Affiliations:** ^1^Service d'Hématologie, CHRU Nancy, rue du Morvan, 54500 Vandoeuvre Les Nancy, France; ^2^Service d'Anatomopathologie, CHRU Nancy, rue du Morvan, 54500 Vandoeuvre Les Nancy, France

## Abstract

We describe a rare case of Castleman disease associated de novo with Hodgkin lymphoma. The incidence of Castleman disease is rare; only a few studies have described it in de novo association with Hodgkin lymphoma. The patient described here complained of unique evolutionary axillary adenopathy. A positron-emission tomography/computed tomography scan revealed hypermetabolic activity in this area. Diagnosis was based on a total excision biopsy of the adenopathy. The patient underwent complete remission with ABVD (doxorubicin, bleomycin, vinblastine, and dacarbazine) chemotherapy for treating Hodgkin lymphoma after surgical excision of the unicentric Castleman disease lesion.

## 1. Introduction

Castleman disease, first described in 1956, is a lymphoproliferative syndrome, also known as angiofollicular lymph node hyperplasia. Its incidence is estimated to be 25 cases per million person per year [1]. We can distinguish two subtypes with very different prognoses. First, Castleman disease in multicentric subtype is often associated with human immunodeficiency virus (HIV) and human herpes virus 8 (HHV-8). A new anti-IL-6 antibody can improve overall survival. In a recent study, the 1-year survival rate was 100% in a group that received siltuximab and 92% in the placebo group [2]. Castleman disease can progress to, or it may be associated de novo with, malignant lymphoma which makes both the diagnosis and treatment complex.

In contrast to multicentric Castleman disease, unicentric Castleman disease is not associated with either HIV or HHV-8 and almost always presents with the hyaline vascular subtype. Surgical resections can provide cures with 10-year overall survival rates greater than 95% [3].

Hodgkin lymphoma (HL) is a lymphoid malignancy characterized by Reed-Sternberg cells within infiltrated lymph nodes. Prognosis and treatment depend on the Ann Arbor stage and patient age.

## 2. Case Presentation

**Figure 1:**
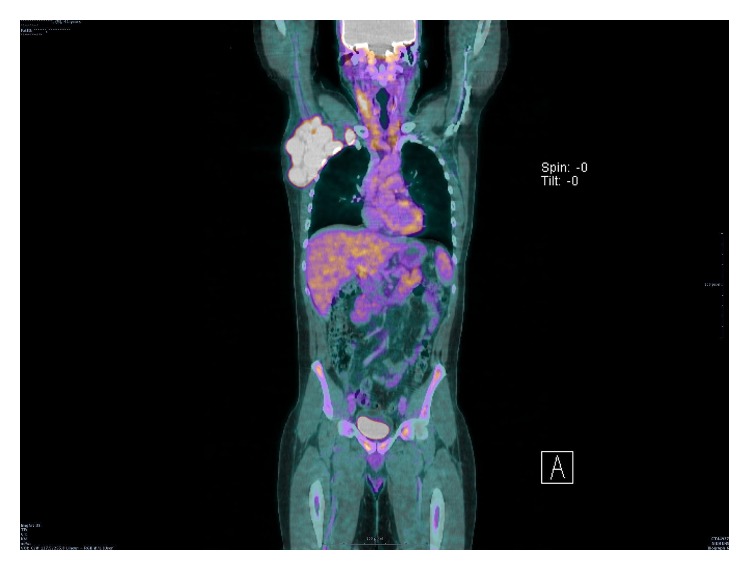
PET CT before treatment. Stage II bulky Hodgkin lymphoma.

**Figure 2:**
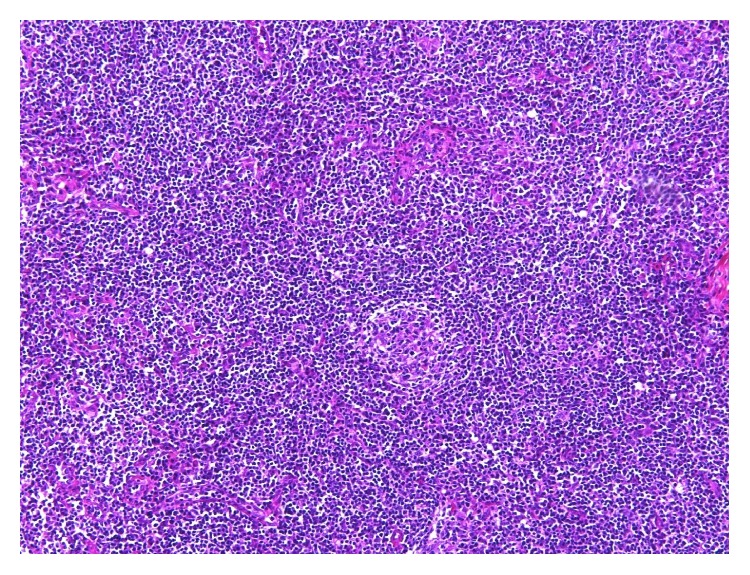
Follicle bulb onion, typical of Castleman disease. Stained section of lymph node biopsy, ×100 magnification, HES coloration.

**Figure 3:**
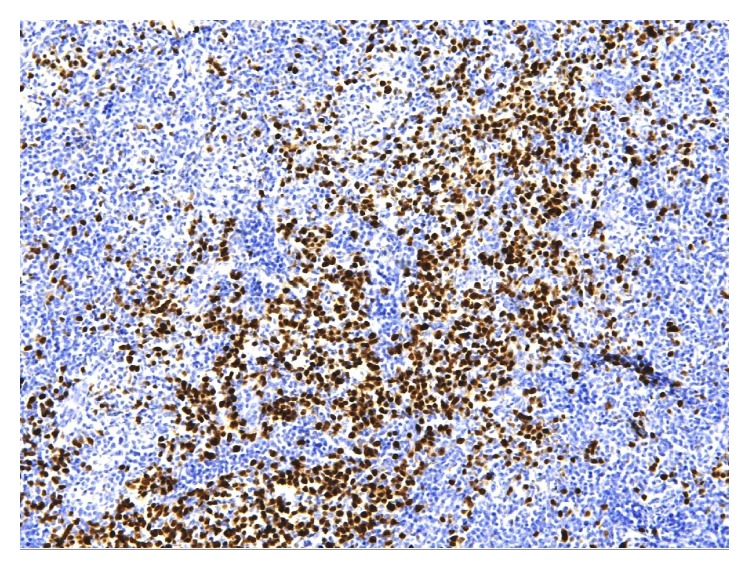
Plasmacytic cells labeled with MUM1 antibody. Section of lymph node biopsy, ×400 magnification.

**Figure 4:**
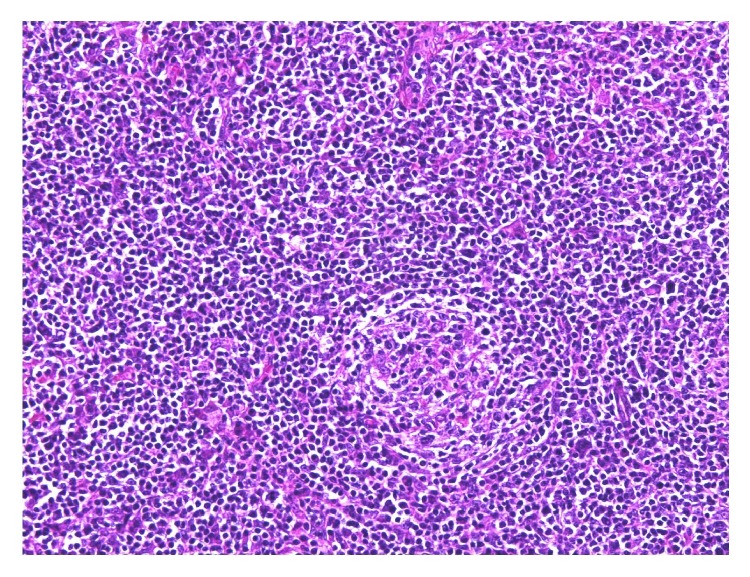
Plasmacytic cell and Hodgkin cell lymphoma. Section of lymph node biopsy, ×100 magnification.

**Figure 5:**
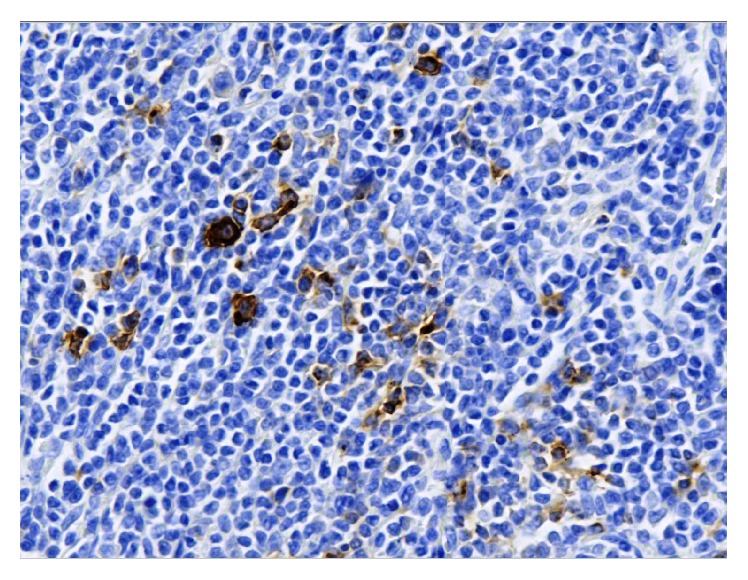
Hodgkin cells stained with anti-CD30 antibodies. Section of lymph node biopsy, ×400 magnification.

**Figure 6:**
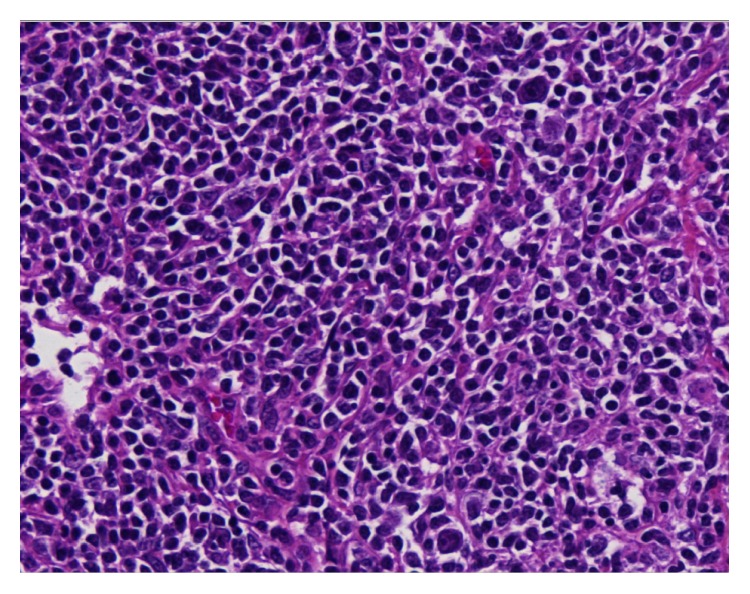
Hodgkin cells. Section of lymph node biopsy, ×400 magnification.

The patient is a 42-year-old Caucasian man with evolving adenopathy. He had not noticed any B symptoms. He was not golfer or hunter and not exposed to forest environment. On June 6, 2016, clinical examination revealed a 10 cm axillary lymphadenopathy without any other tumoral syndrome. He developed normocytic normochromic anemia with hemoglobin count of 12.3 g/dL, platelet count of 506 g/L, leukocyte count of 13.46 Giga/L, and neutrophil count of 11.13 Giga/L. We also noticed signs of inflammatory syndrome with increased erythrocyte sedimentation rate (ESR) 102 mm, high C reaction protein (CRP) 156 mg/L, and hyperferritinemia (624 *μ*g/L). Serum protein electrophoresis revealed polyclonal hypergammaglobulinemia. The lactate dehydrogenase (LDH) was normal (186 UI/L). HIV and HHV-8 serologies were negative. Positron-emission tomography (PET) CT scan showed numerous lymphadenopathy lesions (axillary, pectoral) with a standard uptake value (SUV) max of 10.1 (Figure 1). A macrobiopsy was acquired of the axillary adenopathy. The first histology exam concluded with a diagnosis of Hodgkin lymphoma in lymphocyte rich classic Hodgkin lymphoma variant. A second examination in another laboratory by an expert led to the conclusion that it was a follicular hyperplasia with an atrophic germinal center, with polytypic plasma cells in the interfollicular area; these results suggested that it might be a plasma cell variant of Castleman disease. Immunostaining results were negative for HHV-8 and Epstein Barr virus. We performed a full surgical excision of one of the axillary adenopathies. A new anatomopathologic analysis led to the conclusion that a focal plasma cell variant of Castleman disease (Figures 2 and 3) coexisted in the same interfollicular area as CD30+ HL lesion (Figures 4, 5, and 6). Finally, we diagnosed our patient with stage II A bulky HL, based on the Ann Arbor classification, in lymphocyte rich classic Hodgkin lymphoma variant associated with Castleman disease in unicentric plasma cell variant.

**Figure 7:**
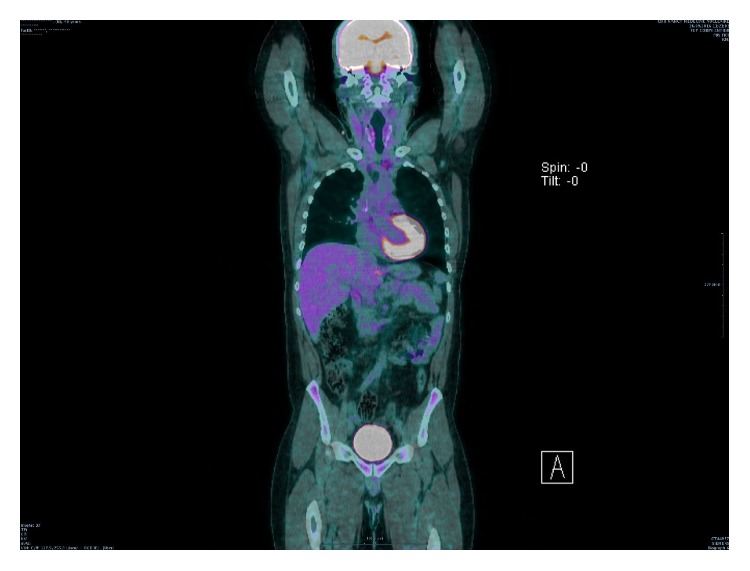
PET CT after treatment.

The patient underwent 4 cycles of chemotherapy with ABVD (doxorubicin, bleomycin, vindesine, and dacarbazine). The patient achieved complete remission, according to the Cheson criteria, assessed with PET CT, after 2 and 4 curative treatments [4] (Figure 7). Next, the patient underwent radiotherapy involving a 30 Gy field exposure completed in 15 sessions, which targeted the site initially involved. On November 29, 2016, the patient achieved complete remission. Follow-up is short to conclude that either disease, Castleman disease or Hodgkin lymphoma, has been cured, but it seems to be in a good way. The patient was included in the French CD registry.

## 3. Discussion

This report describes a rare case of CD associated de novo with HL. Patients with this type of association between two diseases are often negative for HHV-8 and HIV but have a plasma cell variant of CD and an interfollicular variant of HL is present, exactly like our patient. The prognosis of this association seems to be better than prognoses for associations between CD and non-Hodgkin lymphoma. There is no clear explanation for this association. We believe that IL-6 is implicated in the pathogenesis of HL [5]; moreover, it is known that an increase in IL-6 production is part of the pathogenesis of Castleman disease [6]. Plasma cell variant represents less than 10% of Castleman's disease and corresponds to 9% of the cases of localized Castleman's disease. Diagnosis is very difficult in anatomopathology because Reed-Sternberg cells could be dispersed in the biopsy, but they can only be detected with immunochemistry [7]. They localize in interfollicular areas, which can orient incorrectly with respect to reactive hyperplasic follicles. Therefore, in case of CD recurrence, it may be necessary to reexamine the initial biopsy to ascertain the diagnosis. Furthermore, diagnosis is easier with an entire excision than with an echo guided microbiopsy.
